# Supplementary material for: Identification of photoperiod-induced specific miRNAs in the adrenal glands of Sunite sheep (Ovis aries)
Source: Front Vet Sci. 2022 Jul 22;9:888207. doi: 10.3389/fvets.2022.888207 (PMC9354845; doi:10.3389/fvets.2022.888207)
Supplement: Supplementary file 1 [file Data_Sheet_1.ZIP › Supplementary file/miRDeep2 file.zip!/miRDeep2 file/Know_Structure/LP42A1/oar-mir-1185.pdf]

miRBase precursor : oar-mir-1185
Total read count : 147
oar-miR-1185-5p read count : 51
oar-miR-1185-3p read count : 96
remaining reads : 0

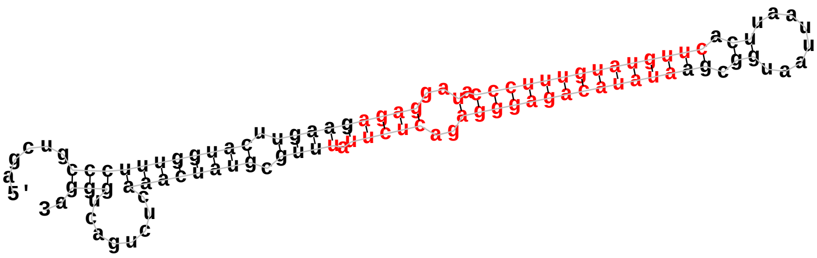

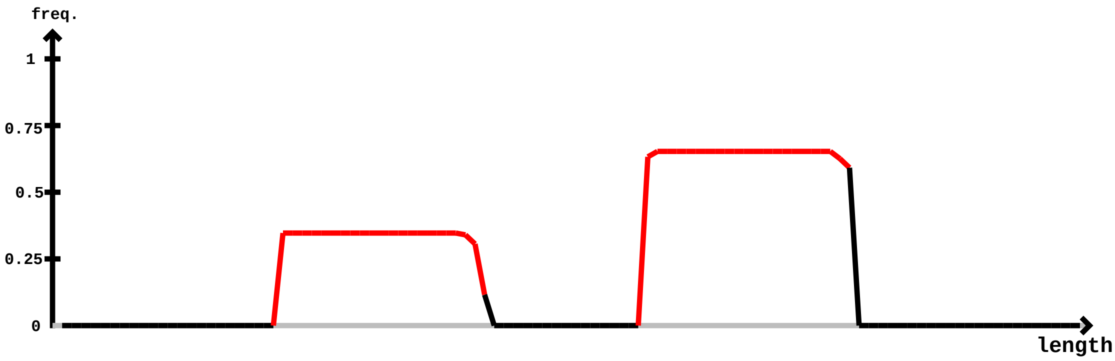

oar-miR-1185-5p

oar-miR-1185-3p

```
5'- agcugcccuuugguacuugaagagaggauacccuuuguauguucacuuaauuaauggcgaauauacagagggagacucuuauuugcguaucaaacucugacuggga   -3'   exp
    .....((((((((((.(((((((((..(.((((((((((((((.((........)).)))))))))))))))..))))).)))).))))))))........))).    reads      mm         sample
    ......................agaggauacccuuuguaug...............................................................    1          0          seq
    ......................agaggauacccuuuguaugu..............................................................    5          0          seq
    ......................agaggauacccuuuguauguu.............................................................    28         0          seq
    ......................agaggauacccuuuguauguuc............................................................    17         0          seq
    ............................................................auauacagagggagacucuu........................    4          0          seq
    ............................................................auauacagagggagacucuua.......................    5          0          seq
    ............................................................auauacagagggagacucuuau......................    84         0          seq
    .............................................................uauacagagggagacucuuau......................    3          0          seq
```
